# Modulation of Autophagy Direction to Enhance Antitumor Effect of Endoplasmic‐Reticulum‐Targeted Therapy: Left or Right?

**DOI:** 10.1002/advs.202301434

**Published:** 2023-06-08

**Authors:** Xinran Shen, Yudi Deng, Liqiang Chen, Chendong Liu, Lian Li, Yuan Huang

**Affiliations:** ^1^ Key Laboratory of Drug‐Targeting and Drug Delivery System of the Education Ministry and Sichuan Province Sichuan Engineering Laboratory for Plant‐Sourced Drug and Sichuan Research Center for Drug Precision Industrial Technology West China School of Pharmacy Sichuan University Chengdu 610041 China

**Keywords:** antimetastasis therapy, autophagy modulation, drug delivery system, endoplasmic reticulum targeting, immunotherapy

## Abstract

Strategies that induce dysfunction in the endoplasmic reticulum (ER) hold great promise for anticancer therapy, but remain unsatisfactory due to the compensatory autophagy induction after ER disruption. Moreover, as autophagy can either promote or suppress cell survival, which direction of autophagy better suits ER‐targeting therapy remains controversial. Here, a targeted nanosystem is constructed, which efficiently escorts anticancer therapeutics into the ER, triggering substantial ER stress and autophagy. Concurrently, an autophagy enhancer or inhibitor is combined into the same nanoparticle, and their impacts on ER‐related activities are compared. In the orthotopic breast cancer mouse model, the autophagy enhancer increases the antimetastasis effect of ER‐targeting therapy and suppresses over 90% of cancer metastasis, while the autophagy inhibitor has a bare effect. Mechanism studies reveal that further enhancing autophagy accelerates central protein snail family transcriptional repressor 1 (SNAI1) degradation, suppressing downstream epithelial–mesenchymal transition, while inhibiting autophagy does the opposite. With the same trend, ER‐targeting therapy combined with an autophagy enhancer provokes stronger immune response and tumor inhibition than the autophagy inhibitor. Mechanism studies reveal that the autophagy enhancer elevates Ca^2+^ release from the ER and functions as a cascade amplifier of ER dysfunction, which accelerates Ca^2+^ release, resulting in immunogenic cell death (ICD) induction and eventually triggering immune responses. Together, ER‐targeting therapy benefits from the autophagy‐enhancing strategy more than the autophagy‐inhibiting strategy for antitumor and antimetastasis treatment.

## Graphical abstract

Therapeutic effects of endoplasmic reticulum (ER)‐targeted therapies are hindered by compensatory autophagy induced by ER disruption. Since autophagy plays dual roles in cell survival, which direction of autophagy better suits ER‐targeted therapy remains unknown. Here, two distinct drug delivery systems are developed to promote (or inhibit) autophagy to investigate potential synergistic effects of autophagy regulators with ER‐targeted therapies.

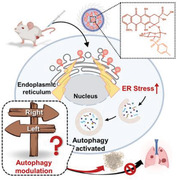

## 1 Introduction

Endoplasmic reticulum (ER) is a vital organelle responsible for protein folding, intracellular homeostasis regulation, and cell proliferation in eukaryotic cells, which establishes ER as a potentially effective target for anticancer therapy.^[^ ^1^, ^2^, ^3^ ^]^ In a previous study, we synthesized a library of doxorubicin (DOX) derivatives with various targeting subunits, including small molecules and short peptides, redirecting the intracellular fate of DOX from nucleus to ER. Our study found that the ER‐targeting efficiency of DOX positively correlated with the capability to induce immunogenic cell death (ICD). Ultimately, we screened out *p*‐toluene sulfonyl‐modified DOX (ED) as the most effective compound, with the highest ER localization and ICD‐inducing ability.^[^ ^4^ ^]^ However, even though ED causes severe ER dysfunction, the cytotoxicity of ED was significantly attenuated. The underlying mechanism could be attributed to our findings that in response to ED‐induced ER stress, autophagy was generated as compensatory and protective mechanism against ED treatment. Therefore, the modulation of autophagy is imperative after effective ER‐targeting therapy.

A new problem has arisen; autophagy is a double‐edged sword playing a dual role in regulating cellular homeostasis.^[^ ^5^, ^6^ ^]^ On the one hand, autophagy could promote cell survival by recycling damaged organelles and misfolded proteins.^[^ ^7^, ^8^ ^]^ Inhibiting autophagy has been shown to activated apoptosis, and restraining the disassembly of metastatic focal adhesion.^[^ ^9^, ^10^ ^]^ For instance, He and co‐workers combined an autophagy inhibitor, chloroquine, with chemotherapeutics to treat breast cancer, and achieved significant antimetastatic effects.^[^ ^11^ ^]^ On the other hand, overactivation of autophagy could trigger the autophagic cell death pathway, which reduces the secretion of metastasis‐related factors and increases the release of ICD‐associated factors in dying cells.^[^ ^12^, ^13^, ^14^ ^]^ Wei and co‐workers, co‐delivering autophagy promoter (rapamycin) and photosensitizer (phthalocyanine) to the tumor, initiated the autophagy process and significantly enhanced the efficacy of photodynamic therapy.^[^ ^15^ ^]^ Nevertheless, on the basis of ER stress‐induced autophagy, which direction to modulate (enhance or inhibit) autophagy better suits ER‐targeting therapy remains unknown.

In this study, two distinct drug delivery systems were developed to either promote or inhibit autophagy in order to investigate the potential synergistic effects of autophagy regulators with ER‐targeted therapies. For this purpose, ED was selected as an ER stress inducer. The *p*‐toluene sulfonyl guided ED to sulfonamide receptors on ER and was decomposed by carboxylesterases, resulting in the release of DOX from ED.^[^ ^4^, ^16^, ^17^ ^]^ It was reported that 3‐methyladenine could inhibit the formation of a double membrane, named phagophore, thereby suppressing the formation of autophagosome.^[^ ^18^, ^19^ ^]^ Thus, we denoted 3‐methyladenine as the autophagy inhibitor (AP^I^). Rapamycin was reported to induce autophagosome formation and the subsequent fusion of lysosomes and autophagosomes.^[^ ^20^, ^21^ ^]^ so that we denoted rapamycin as an autophagy enhancer (AP^E^). Hence, AP^I^ or AP^E^ were co‐loaded with ED in Poly‐lactic‐*co*‐glycolic acid (PLGA) nanoparticle (NP), respectively. ICD inducing and immune activating performance of different autophagy modulators with ED was compared, and the antitumor effects were subsequently investigated. In parallel, the metastasis suppression effects and the underlying mechanisms of the two different strategies were elucidated. Collectively, our research provided a systematic study of the impact of regulating autophagy on ER‐targeted therapies, including differences in their antitumor and antimetastasis effects, which might shed light on further studies on efficient antitumor and antimetastasis therapies.

## 2 Results and Discussion

### 2.1 Both Autophagy Activation and Inhibition Potentiate the Cytotoxicity of ED

**Figure 1:**
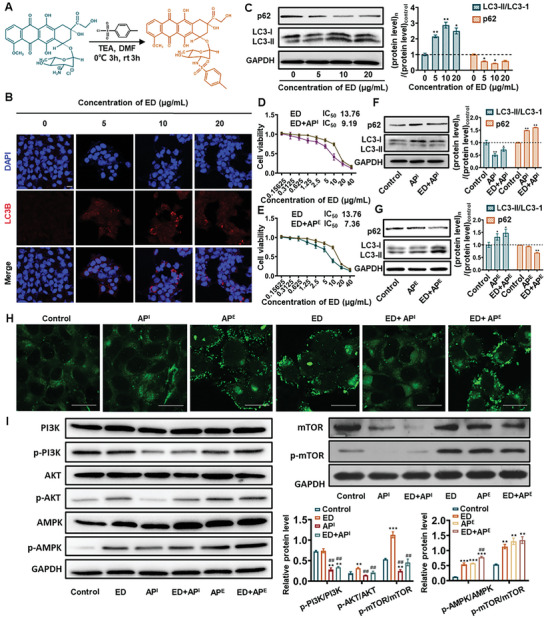
A) The synthesis route of ED. B) Confocal laser scanning microscopy (CLSM) images of accumulated autophagosomes (red spots) in ED‐treated 4T1 cells after 24 h (scale bar = 20 µm). C) The protein level of the autophagic substrate (LC3B) analyzed in 4T1 cells treated with ED at different concentrations for 24 h by western blotting. D) Cell viability curves of AP^I^ (70 µm) and E) AP^E^ (10 µm) in combination with a series concentrations of ED on 4T1 cells for 24 h, respectively. F) The protein level of LC3 analyzed in 4T1 cells after AP^I^ and (ED+ AP^I^) treatment by western blotting. G) The protein level of LC3B analyzed in 4T1 cells after AP^E^ and (ED+ AP^E^) treatment by western blotting. H) LC3 punctate dots in different treatment groups in 4T1 cells (scale bar = 20 µm). I) The protein level of PI3K, p‐PI3K, AKT, p‐AKT, AMPK and p‐AMPK, mTOR, and p‐mTOR in 4T1 cells after treatment for 24 h by western blotting. All results were presented as mean ± standard deviation (SD). *n* = 3. *p* ^*^<0.05, *p* ^**^<0.01, *p* ^***^<0.001 versus control. *p* ^##^<0.01 versus ED.

First, the ER stress inducer ED was synthesized (**Figure** **1**A) and confirmed by ^1^H‐NMR and mass spectrum (Figure S1, Supporting Information). Subsequently, various concentrations of ED (5, 10, and 20 µg mL^−1^) were selected to assess the autophagy induction ability in 4T1 cells. As shown in Figure 1B, all of ED‐treated groups formed red spots that represented autophagosomes comparing with the untreated group. Besides, the results from Figure 1C revealed that ED promoted the upregulation of LC3‐II, which could be attributed to cytoplasmic hydrolyzed LC3‐I converting to LC3‐II that appearing on the autophagosome membrane.^[^ ^22^ ^]^ In addition, ED promoted p62 degradation, suggesting that autophagy process was activated.

To regulate autophagy to two different directions, the autophagy enhancer AP^E^ (rapamycin) or the autophagy inhibitor AP^I^ (3‐methyladenine) was selected at the concentrations of 10 and 70 µm, respectively (Figure S2, Supporting Information). At the selected concentrations, the two autophagy modulators had minor toxicity to 4T1 cells, with the cell viability of 87.2% and 90.1%, respectively. As shown in Figure S3 (Supporting Information), AP^E^ or AP^I^ could remarkably enhance or inhibit autophagy, suggesting that both regulators were sufficient to trigger the autophagy‐regulating effect.

After determining the appropriate concentrations of autophagic modulators, the combined effects of ED with different autophagy modulators were evaluated. As shown in Figure 1D,E, when AP^E^ or AP^I^ was combined with ED (ED+AP^E^ or ED+AP^I^), the cytotoxicity of both ED+AP^E^ and ED+AP^I^ was strongly enhanced in 4T1 cells compared to ED with the half maximal inhibitory concentration (IC_50_) of 13.76 µg mL^−1^. Nevertheless, the two combination strategies both increased the cytotoxicity of ED, with the IC_50_ of ED+AP^E^ and ED+AP^I^ at the same order of magnitude (IC_50_ = 7.36 and 9.19 µg mL^−1^). In terms of the ability to modulate autophagy, the downregulated LC3‐II/ LC3‐I ratio and upregulated p62 expression suggested that ED+AP^I^ inhibited cellular autophagy (Figure 1F), whereas ED+AP^E^ did the opposite (Figure 1G). Furthermore, immunofluorescence results further confirmed the autophagy‐modulating ability of ED+AP^I^ and ED+AP^E^ (Figure 1H). (ED+AP^E^)‐treated cells presented more green punctate dots, indicating that more LC3 proteins were accumulated on autophagosome membranes and autophagy process was stimulated. In contrast, barely no obvious green punctate dots were observed in (ED+AP^I^)‐treated cells, confirming its autophagy inhibiting ability. The above results indicated that the combination of two autophagy modulators with ED had comparable cytotoxicity. Therefore, subsequent differences in the results obtained from using the two combined therapies would only be attributed to the different directions of regulating autophagy.

The intrinsic mechanism regulating autophagy was then investigated. The available research suggests that PI3K/AKT/mTOR (Phosphoinositide 3‐kinase/Protein kinase B/Mammalian target of rapamycin) and AMPK/mTOR (Adenosine 5‘‐monophosphate ‐activated protein kinase/Mammalian target of rapamycin) pathways both modulate autophagy via mTOR signaling, and these two pathways are also essential pro‐survival mechanisms when cells respond to stress.^[^ ^23^, ^24^ ^]^ AMPK and AKT, as two major effectors of the two pathways, generally function as antagonistic roles under stress, and jointly regulate mTOR to maintain adaptive autophagy level.^[^ ^25^, ^26^, ^27^ ^]^ As shown in Figure 1I, compared to the ED‐treated group, the ratio of *p*‐PI3K/ PI3K, *p*‐AKT/AKT, and *p*‐mTOR/mTOR in AP^I^‐ and (ED+AP^I^)‐treated cells markedly downregulated, demonstrating that AP^I^ blocked the autophagy process by PI3K/AKT/mTOR pathway. Moreover, the upregulated ratio of *p*‐AMPK/AMPK and *p*‐mTOR/mTOR in AP^E^‐ and (ED+AP^E^)‐treated cells relative to the ED‐treated group suggested AP^E^ promoting the autophagy process by AMPK/mTOR pathway. The above results demonstrated that the two compensatory prosurvival pathways were activated in cells after ED treatment, while both autophagy enhancement and inhibition disrupted these compensatory pathways and increased cell apoptosis.

### 2.2 Construction of Drug Delivery System for Codelivery of ED and Autophagy Modulators

**Figure 2:**
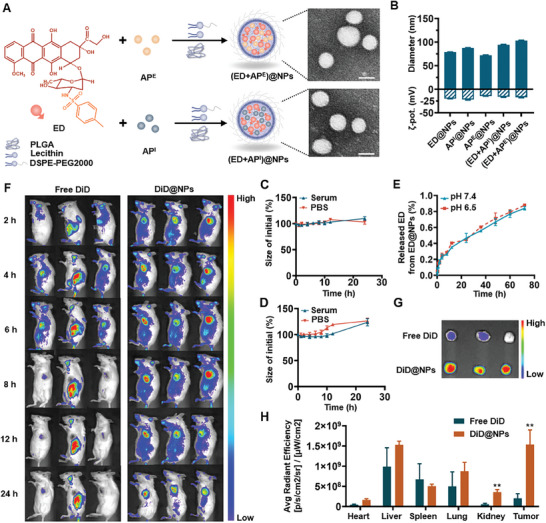
A) Schematic illustration of nanoparticles and representative image of (ED+AP^E^)@NPs and (ED+AP^I^)@NPs examined with transmission electron microscopy (scale bar = 50 nm). B) Size and zeta potential of nanoparticles of all groups with the diagrams of nanoparticles. C) The release profile of ED@NPs in various pH conditions. D) Size changes of (ED+AP^I^)@NPs or E) (ED+AP^E^)@NPs in PBS (pH 7.4) and serum during 72 h incubation. F) Real‐time in vivo fluorescence intensity of dissected 4T1 tumors in mice at 2, 4, 6, 8, 12, and 24 h after injection of free DiD or DiD@NPs. G) Ex vivo fluorescence intensity of tumors at 24 h and H) mean fluorescence intensity of major organs and tumors. All results were presented as mean ± SD. *n* = 3. *p* ^**^<0.01 versus Free DiD.

To achieve superior in vivo targeting ability, a nanodrug delivery system was developed for co‐encapsulation of ED and autophagy regulators. PEG (Polyethylene glycol)–PLGA nanoparticles containing both ED and autophagy regulators were manufactured using the nanoprecipitation method, and the transmission electron microscopy images showed that both (ED+AP^E^)@NPs and (ED+AP^I^)@NPs were spherical in shape (**Figure** **2**A).^[^ ^27^ ^]^ All nanoparticles were spherical with sizes ranging from 70 to 100 nm and negatively charged surfaces (Figure 2B). And the final prescription insured the same dose ratio as the previous combination strategies, with the equivalence DOX dose of 5 µg mL^−1^ and equivalence AP^I^ dose of 70 µm or equivalence AP^E^ dose of 70 µm, and the mass ratio of ED and AP^I^ (AP^E^) were ED:AP^I^ = 1:0.75 and ED:AP^E^ = 1:0.457, respectively (Table S1, Supporting Information). The results in Figure 2B demonstrated a sustained drug release profile under both pH conditions (pH 6.5 and 7.4). After incubation in phosphate‐buffered saline (PBS, pH 7.4) or serum for 48 h, no significant changes in nanoparticles size were observed, indicating excellent physiological stability (Figure 2C–E).

Next, the tumor‐targeting capacity of nanoparticles was validated in vivo. After administration via the tail vein, as shown in Figure 2F, free DiD (1,1'‐Dioctadecyl‐3,3,3',3'‐Tetramethylindodicarbocyanine,4‐Chlorobenzenesulfonate Salt) was rapidly distributed to the liver and tumor at 2 h after injection, gradually distributed throughout the body within 4–8 h and then weakened, indicating that free DiD was easily and rapidly cleared in vivo. Conversely, DiD@NPs exhibited persistently high fluorescence at the tumor site during 24 h, demonstrating that nanoparticles could passively accumulate at the tumor site and prolong the retention in the tumor. According to the ex vivo mean fluorescence intensity results at 24 h, the fluorescence intensity of DiD@NPs in tumor tissues was 7.5 times that of free DiD (Figure 2G,H; Figure S4, Supporting Information). The findings indicated that nanoparticles have excellent tumor‐targeting and tumor‐accumulating abilities.

### 2.3 Metastasis Suppression: AP^I^ versus AP^E^

**Figure 3:**
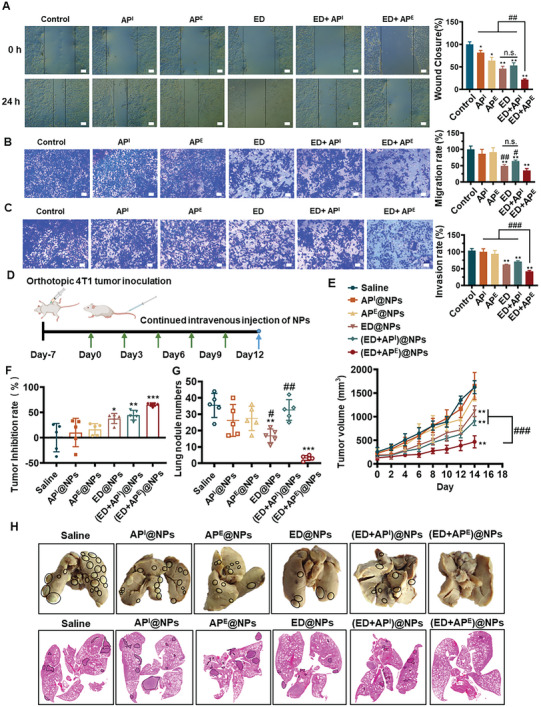
Images of A) wound healing, B) migration, and C) invasion of 4T1 cells after treatment for 24 h, *n* = 3 (scale bar = 200 µm). D) Schematic diagram of the in vivo antimetastatic study model establishment and dosing regimen (*n* = 5). E) Tumor growth curves of mice (*n* = 5). F) Tumor inhibition rate of mice in each group (*n* = 5). G) Lung metastatic nodule numbers after day 21 of tumor inoculation and H) representative pictures of lungs in each group (*n* = 5). All results were presented as mean ± SD. *p* ^*^< 0.05, *p* ^**^ < 0.01, *p* ^***^ < 0.001, n.s.: not significant versus saline. *p* ^#^ < 0.05, *p* ^##^ < 0.01, *p* ^###^ < 0.001 versus (ED+AP^E^).

Research to date reveals that the development of autophagy might alter tumor metastasis processes, especially during the stage when tumor cells separate from the primary tumors.^[^ ^28^ ^]^ Specifically, the level of autophagy might affect tumor–stroma interactions and consequently initiate or restrict metastatic spread.^[^ ^29^ ^]^ Therefore, wound‐healing assay, cell migration, and invasion assays were performed to assess how the two distinct strategies influence tumor metastasis processes. All the chosen concentrations of the drugs ensured their cell viability results to above 80% (Figure S5, Supporting Information). As shown in **Figure** **3**A, cells treated with ED+AP^E^ and ED had smaller healing regions compared to cells treated with ED+AP^I^. In cell migration assay (Figure 3B), ED+AP^I^ was able to significantly diminish the migration of 4T1 cells with a migration rate of 64.13%. In comparison, both ED and ED+AP^E^ outstandingly inhibited the migration of 4T1 cells, with migration rates of 49.51% and 35.59%, respectively. Only ED+AP^E^ had significantly stronger migration inhibitory effects as compared with ED. ED+AP^E^ (vs ED and ED+ AP^I^) also exhibited stronger ability to inhibit tumor cell invasion with the relative invasion rate decreasing to 41.98% (Figure 3C), validating that ED+AP^E^ most significantly prevented cancer cells from spreading.

We next investigated in vivo antimetastasis effects of both combination strategies. The administrated drugs were kept the same mass ratios as in vitro experiments (ED:AP^I^ = 1:0.75 and ED:AP^E^ = 1:0.457), with equivalent drug concentrations of 5 µmol kg^−1^ DOX, 122 µmol kg^−1^ AP^I^, and 17.4 µmol kg^−1^ AP^E^, respectively. After 7 days of tumor inoculation, each group of mice received different nanoparticles via the tail vain every 3 days (Figure 3D). As displayed in Figure 3E,F, (ED+AP^E^)@NPs exhibited the most potent antitumor ability (Figure S6, Supporting Information). Moreover, the lung metastatic nodules were counted after 21 days of tumor inoculation. Numerous metastatic nodules occurred in the saline group, and many metastatic nodules were also discovered in the single autophagy modulator groups, demonstrating that it was challenging to inhibit tumor metastasis in vivo by regulating autophagy a lone (Figure 3G,H). The (ED+AP^E^)@NPs group had minimal metastatic nodules and the highest antimetastatic efficacy. On the contrary, the (ED+AP^I^)@NPs group was less effective in metastasis than the ED@NPs group, suggesting that suppression of autophagy might diminish the in vivo antimetastatic efficacy of ED. These findings indicated that autophagy activation reinforced ED in suppressing tumor metastasis.

### 2.4 Mechanism Study for AP^E^ > AP^I^ on Metastasis Inhibition

**Figure 4:**
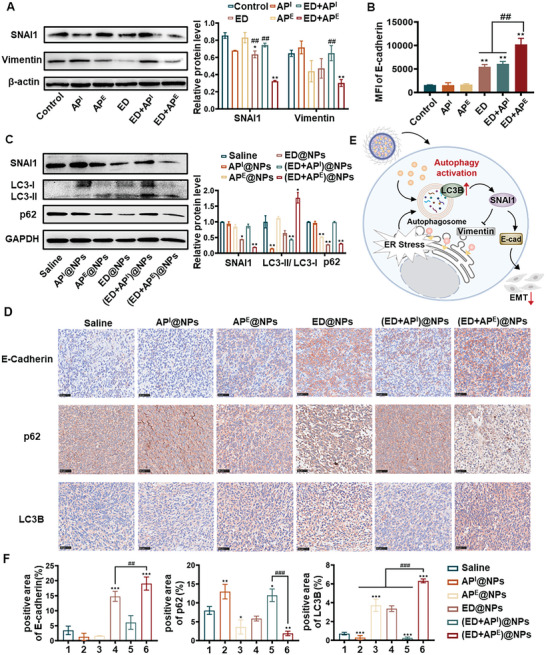
A) The protein level of SNAI1 and vimentin in different treatment groups in 4T1 cells by western blotting. B) Flow cytometry detection of E‐cadherin expression in 4T1 cells after different treatments for 24 h. C) The protein level of p62, LC3B, SNAI1, and vimentin in 4T1 tumors in mice by analyzed western blotting. D) Representative immunohistochemical images of tumor tissues of each group stained for E‐cadherin, p62, and LC3B (scale bar = 50 µm). E) The schematic illustration of the mechanisms of the autophagy enhancer suppressing tumor metastasis. F) The semiquantitative analysis results of tumor tissues immunohistochemical images in each group stained for E‐cadherin, p62, and LC3B (*n* = 3). All results were presented as mean ± SD. *n* = 5. *p* ^*^ < 0.05, *p* ^**^ < 0.01 versus saline. *p* ^##^ < 0.01, *p* ^###^ < 0.01 versus (ED+AP^E^).

Thereafter, it was speculated that ED+AP^E^ would rely on autophagy activation to suppress tumor metastasis. Previous studies have demonstrated that autophagy regulated numerous metastatic factors, thereby affecting several discrete steps throughout the migration process of tumor cells from the primary site to a distant location.^[^ ^30^, ^31^ ^]^ Therefore, the role of autophagy in the regulation of epithelial–mesenchymal transition (EMT) and metastasis‐related proteins under ER‐targeted treatment was investigated.^[^ ^32^, ^33^ ^]^ It was discovered that the levels of snail family transcriptional repressor 1 (SNAI1), an EMT‐inducing transcription factor controlling the initiation of EMT, were significantly decreased when autophagy was induced by ED+AP^E^ in 4T1 cells (**Figure** **4**A).^[^ ^34^, ^35^ ^]^ In comparison, SNAI1 degradation was significantly decreased by the addition of ED+AP^I^. Moreover, the level of epithelial protein E‐cadherin was highly increased by activating autophagy after treatment with ED+AP^E^ (Figure 4B), while there was a large decrease in the level of vimentin (Figure 4A).

Moreover, the expressions of metastasis‐related proteins and autophagy‐associated proteins in tumor tissues were also examined by western blotting and immunohistochemistry (Figure 4C,D,F). SNAI1 and p62 were most significantly degraded in the (ED+AP^E^)@NPs group (vs ED@NPs group), and the proportion of LC3B‐II was significantly higher, indicating that autophagy was greatly stimulated in these two groups and verifying the selective autophagic degradation of SNAI1. In addition, only the treatment of ED@NPs and (ED+AP^E^)@NPs led to an increase in E‐cadherin, indicating that tumor metastasis was suppressed. In contrast, the outcomes of AP^I^@NPs and (ED+AP^I^)@NPs were significantly different. In these two groups, reduction in E‐cadherin, accumulation of p62, and degradation of LC3B‐II were discovered, suggesting a situation that was more conducive to tumor metastasis.

In conclusion, ED+AP^E^ had a stronger ability to dampen tumor cell migration and invasion (vs ED+ AP^I^) both in vitro and in vivo, revealing that autophagy activation reinforced the antimetastatic capacity of ED. ED+AP^E^ also increased autophagy‐dependent SNAI1 degradation, which acts as a major driver of EMT and subsequently regulated the expression of EMT‐related proteins such as E‐cadherin and vimentin, indicating the blocked EMT process. In contrast, AP^I^ and ED+ AP^I^ did not affect SNAI1 degradation. These results indicated that for ER stress‐induced autophagy, enhancing autophagy accelerated SNAI1 degradation, resulting in the downregulation of vimentin and the overexpression of E‐cadherin, suggesting the termination of the EMT process (Figure 4E). In general, the major ER‐related metastasis process was tightly tied to SNAI1‐related autophagy pathway.

### 2.5 Tumor Inhibition: AP^I^ versus AP^E^

**Figure 5:**
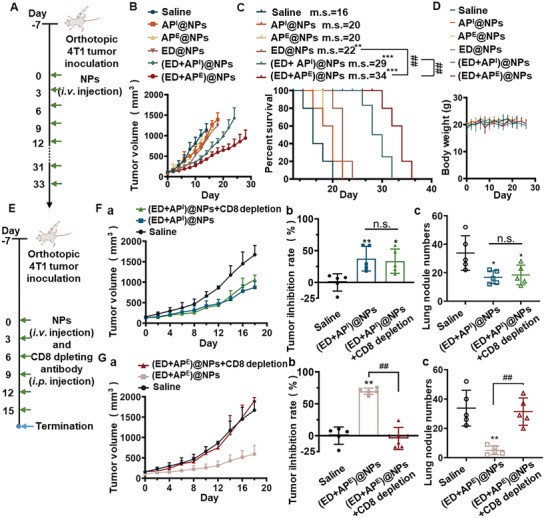
A) Schematic illustration of model establishment and dosing regimen for in vivo antitumor study. B) Tumor growth curves of 4T1 tumor‐bearing balb/c mice treated with different nanoparticles. C) Survival curves and median survival of 4T1 tumor‐bearing balb/c mice treated with different nanoparticles. D) Weight changes of mice during antitumor treatment. E) Schematic diagram of the CD8 depletion model establishment and dosing regimen. F‐a) Tumor growth curves and b) tumor suppression rates with c) the lung metastatic nodule numbers of mice after treatments of (ED+AP^I^)@NPs, (ED+AP^I^)@NPs with CD8 depleting antibodies. G‐a) Tumor growth curves and b) tumor suppression rates with c) the lung metastatic nodule numbers of mice after treatments of (ED+AP^E^)@NPs, (ED+AP^E^)@NPs with CD8 depleting antibodies. All results were presented as mean ± SD. *n* = 5. *p* ^*^ < 0.05, *p* ^**^ < 0.01, *p* ^***^ < 0.001, n.s.: not significant, versus saline. *p* ^##^ < 0.01 versus (ED+AP^E^).

In light of the observation that both autophagy modulators comparably enhanced the in vitro cytotoxicity of ED, further investigation was conducted to evaluate the in vivo tumor‐killing efficacy of the two combined groups. The balb/c mice were subcutaneously implanted with a 4T1 cell to evaluate the in vivo antitumor efficacy of different nanoparticles (**Figure** **5**A). The administrated drugs were kept the same mass ratios as the in vitro experiments. All mice in the saline group experienced exponential tumor growth and quick death, while the (ED+AP^I^)@NPs‐ and (ED+AP^E^)@NPs‐treated groups showed potent antitumor ability with prolonged median survival of mice to 29 and 34 days, respectively (Figure 5B,C). Besides, H&E (Hematoxylin‐Eosin) staining of major organs (Figure S7, Supporting Information) and the body weight of mice (Figure 5D) suggested a good safety profile of nanoparticles. Subsequently, nanoparticles were administered alongside the CD8 depletion antibody in 4T1 tumor‐bearing mice (Figure 5E). As shown in Figure 5F, the tumor growth curve of the (ED+AP^I^)@NPs with CD8 depletion antibody‐treated group and the (ED+AP^I^)@NPs group shared the same trend, so as the number of pulmonary metastatic nodules. In contrast, the CD8 depletion antibody in (ED+AP^E^)@NPs group potently accelerated tumor growth and obviously increased numbers of metastatic lung nodules compared with (ED+AP^E^)@NPs group (Figure 5G), which might be attributed to the fact that the antitumor capacity of (ED+AP^E^)@NPs strongly depend on tumor immune response.

### 2.6 Mechanism Study for AP^E^ > AP^I^ on Tumor Inhibition

**Figure 6:**
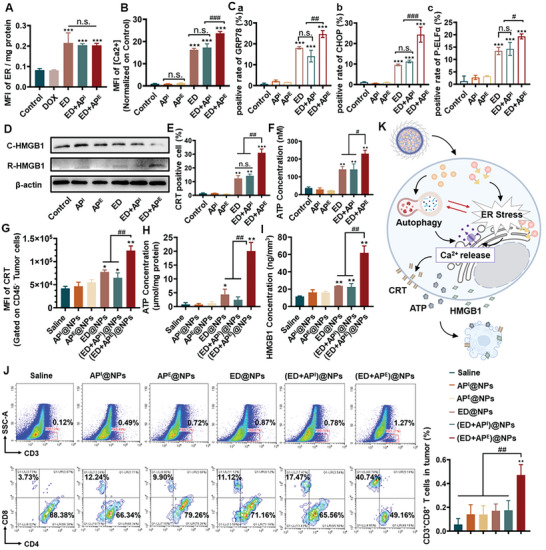
A) The accumulation of ED in 4T1 cells after treatments for 2 h. The MFI (Mean fluorescence intensity) of ER was normalized by total protein concentrations. B) Determination of intracellular Ca^2+^ level in 4T1 cells after treatments for 12 h. C) Flow cytometry analysis of intracellular a) GRP78, b) CHOP, and c) P‐ELF2*α* expressions. D) The protein level of HMGB1 inside 4T1 cells (C‐HMGB1) and in the culture medium (R‐HMGB1) after treatments. E) Flow cytometry analysis of surface CRT in 4T1 cells after treatments for 24 h. F) ATP levels in the culture medium of 4T1 cells after treatments for 24 h. G) Flow cytometry analysis of CRT exposure on CD45^−^ tumor cells. H) Determination of ATP secreted into tumor milieu. I) HMGB1 levels in tumor tissues. J) Representative flow cytometry plots and quantitative analysis of CD3^+^ CD8^+^ T cells in tumors. K) Schematic illustration of the mechanisms for the autophagy enhancer enhancing ICD. All results were presented as mean ± SD. *n* = 5. *p* ^*^ < 0.05, *p* ^**^ < 0.01, *p* ^***^ < 0.001, n.s.: not significant, versus saline. *p* ^#^ < 0.05, *p* ^##^ < 0.01, *p* ^###^ < 0.001 versus (ED+AP^E^).

In vivo studies have revealed that ED+AP^E^ exhibited superior efficacy in inhibiting tumor growth. As a result, further investigation was conducted to elucidate the underlying mechanisms responsible for the enhanced antitumor ability conferred by the autophagy enhancer. Initially, the accumulation of ED in ER was nearly the same across ED and ED‐coupled autophagy regulator groups, indicating that the levels of ED accumulated in ER were insusceptible by autophagy regulators (**Figure** **6**A). Thereafter, whether the autophagy enhancer alters ER function was verified. During ER stress, Ca^2+^ would release from the lumen of ER into the cytoplasm.^[^ ^36^, ^37^ ^]^ As shown in Figure 6B, further increased intracellular calcium level was observed in the ED+AP^E^ group comparing to ED. Meanwhile, the expression of representative ER stress‐related proteins (P‐elf2*α*, CHOP, and GRP78) was examined. As shown in Figure 6C, ED was capable of increasing the expression of ER stress‐related proteins, which may be attributed to its ability to cause ER damage and subsequent ER stress. Moreover, the expression of the proteins rose substantially after AP^E^+ED treatment, implying stronger ER stress. The above results suggested that AP^E^ elevated Ca^2+^ release from ER and increased ER stress.

Our previous research demonstrated that ED may cause cellular ICD; however, the ICD‐inducing potential of ED after modulating autophagy remains unknown. The results in Figure 6D–F showed that the ED+AP^E^ group significantly increased the CRT (Calreticulin) exposure of 4T1 cells, as well as extracellular ATP level and HMGB1 (High mobility group protein) level, implying the strongest ICD induction ability of the ED+AP^E^ group. In contrast, the ICD induction ability of the ED+AP^I^ group had no significant difference compared to that of the ED group, indicating that the ICD induction ability of ED could be greatly improved only with the combination of the autophagy enhancer. In vivo ICD‐inducing capacity of the nanoparticles was subsequently evaluated, displaying a similar trend. ED@NPs and (ED+AP^E^)@NPs were able to obviously stimulate CRT expression from ER to the cell membrane surface, as shown in Figure 6G, while no significant increase in CRT expression in other groups. The results in Figure 6H demonstrated that the ATP release was 23 times greater in the (ED+AP^E^)@NPs group than in the ED@NPs group, which was presumably the consequence of the autophagy‐dependent ATP release based on ED treatment.^[^ ^38^ ^]^ Also, the HMGB1 level in tumor interstitial fluid (Figure 6I) revealed that the (ED+AP^E^)@NPs group was 5‐fold higher than the saline group and 2.7‐fold greater than the ED@NPs group. The aforementioned results demonstrated that the increased levels of Ca^2+^ and ER stress resulting from enhanced autophagy further amplified ED‐induced ICD.

Considering that autophagy mediates the release of tumor antigens and danger‐associated molecular patterns (DAMP), enhancing autophagy might restrain advanced tumors from escaping immunosurveillance.^[^ ^39^, ^40^, ^41^ ^]^ Thereby, ED+AP^E^ might contribute to mounting antigen‐specific T‐cell responses we speculated.^[^ ^42^ ^]^ The role of T lymphocytes was investigated. Notably, the infiltration of CD3^+^ T cells in tumors and the proportion of CD8^+^ T cells among them were elevated after treatment with NPs (Figure 6J). However, only (ED+AP^E^)@NPs resulted in a significant increase in the proportion of cytotoxic CD3^+^CD8^+^ T cells, 8.47 times more than saline. These results indicated that (ED+AP^E^)@NPs could boost intense ICD, thereby leading to a robust tumor immune response.

In summary, we verified that AP^E^ did not affect the accumulation of ED in ER. Then we were surprised to discover that the levels of ER stress‐related proteins were considerably upregulated by ED+AP^E^, demonstrating AP^E^ could magnify ED‐induced ER stress. Moreover, the Ca^2+^ released in the cytoplasm was dramatically elevated by ED+AP^E^, suggesting that AP^E^ might increase the release of Ca^2+^ via amplifying ER stress. Correspondingly, our results showed that only ED+AP^E^ was capable of augmenting ED‐induced ICD. Besides, only ED+AP^E^ intensively increased the tumor infiltration of CD8^+^ T cells and led to robust immune response, whereas the antitumor process of ED+AP^I^ had little involvement of CD8^+^ T cells. Consequently, enhancing autophagy‐elevated intracellular Ca^2+^ release from ER, and functioned as an amplifier of ER dysfunction→Ca^2+^ release → ICD induction → immune response cascade, eventually eliciting powerful antitumor effect (Figure 6K). In contrast, inhibiting autophagy failed to affect intracellular Ca^2+^ levels, and only increased apoptosis by disrupting compensatory prosurvival pathways (Figures 1I and 6B).

**Scheme 1:**
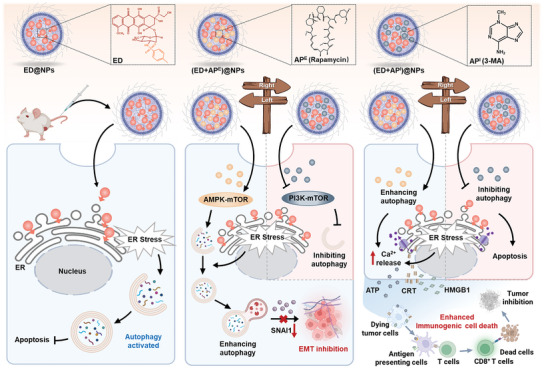
Schematic illustration of the autophagy‐modulating drug delivery strategies. ER‐targeted ED could induce ER stress, but compensatory autophagy was also activated and decreased its cytotoxicity. ED and autophagy modulators (AP^E^ or AP^I^) were co‐loaded into nanoparticles. (ED+AP^E^)@NPs accelerated the degradation of central protein SNAI1, leading to the suppression of the downstream metastasis‐promoting process of EMT. In parallel, (ED+AP^E^)@NPs functioned as an amplifier of ER dysfunction → Ca^2+^release → ICD induction → immune response cascade, provoking stronger immune response. However, (ED+AP^I^)@NPs only promoted apoptosis via blocking the PI3K/mTOR pathway.

Intriguingly, it is reported that the escalation of intracellular Ca^2+^ release into the cytoplasm under ER stress conditions might also promote autophagy, which is attributed to the activation of the CamKK/AMPK‐dependent pathway by released Ca^2+^.^[^ ^43^, ^44^ ^]^ Then it could be speculated that in the (ED + AP^E^)‐treated group, ED upregulated Ca^2+^ level and activated autophagy while AP^E^ could increase Ca^2+^ release via enhancing autophagy. In this way, AP^E^ functioned as an amplifier in ER stress‐Ca^2+^ release network, eventually resulting in robust ER stress and extremely elevated Ca^2+^ release, thus triggering robust ICD. In contrast, inhibiting autophagy did not affect intracellular Ca^2+^ levels, so it failed to affect ER stress‐Ca^2+^ release network (**Scheme** **1**).

## 3 Conclusion

In summary, this study investigated the influences of autophagy modulation on ER‐targeted therapies in antitumor metastasis and immune activation. It was discovered that ER‐targeting therapy benefits from autophagy‐enhancing strategy more than autophagy‐inhibiting strategy for antitumor and antimetastasis treatment. The ER‐related downstream activities and the underlying mechanisms were elucidated: 1) the main ER‐associated metastasis process was tightly tied to SNAI1‐related autophagy pathway, and enhancing autophagy consecutively activated SNAI1 degradation, and the blocked EMT process, leading to much higher metastasis suppression as compared with ED; and 2) enhancing autophagy‐elevated Ca^2+^ release from ER and functioned as an cascade amplifier of ER dysfunction, which subsequently increased Ca^2+^ release, resulted in ICD induction and eventually triggered immune response. Collectively, enhancing autophagy could synergistically improve antitumor capability by preventing tumor metastasis and promoting ICD‐associated immunotherapy on the basis of ER stress induction. Meanwhile, the approach of combining ER‐targeted system and autophagy enhancing strategy could serve as a generalized framework to strengthen the therapeutic effects and provide an option for efficient antitumor as well as antimetastasis therapies.

## 4 Experimental Section

The full materials and experimental methods can be found in the Supporting Information. All of the animal experiments were approved by the Medical Ethics Committee of Sichuan University, and the animal experiments were performed in the Animal Laboratory of West China School of Pharmacy in Sichuan University (accreditation number: SYXK(Chuan)2018‐113).

## Conflict of Interest

The authors declare no conflict of interest.

## Supporting information

Supporting Information

## Data Availability

The data that support the findings of this study are available from the corresponding author upon reasonable request.
