# Supplementary material for: Modulation of Autophagy Direction to Enhance Antitumor Effect of Endoplasmic‐Reticulum‐Targeted Therapy: Left or Right?
Source: Adv Sci (Weinh). 2023 Jun 8;10(23):2301434. doi: 10.1002/advs.202301434 (PMC10427372; doi:10.1002/advs.202301434)
Supplement: Supplementary file 1 — Supporting Information [file ADVS-10-2301434-s001.pdf]

ADVANCED SCIENCE
Open Access

Supporting Information

for *Adv. Sci.*, DOI 10.1002/advs.202301434

Modulation of Autophagy Direction to Enhance Antitumor Effect of Endoplasmic-Reticulum-Targeted Therapy: Left or Right?

Xinran Shen, Yudi Deng, Liqiang Chen, Chendong Liu, Lian Li and Yuan Huang**

Supporting Information

Modulation of Autophagy Direction to Enhance Anti-tumor Effect of Endoplasmic Reticulum-targeted Therapy: Left or Right?

Xinran Shen, Yudi Deng, Liqiang Chen, Chendong Liu, Lian Li, Yuan Huang**

Key Laboratory of Drug-Targeting and Drug Delivery System of the Education Ministry and Sichuan Province, Sichuan Engineering Laboratory for Plant-Sourced Drug and Sichuan Research Center for Drug Precision Industrial Technology, West China School of Pharmacy, Sichuan University, Chengdu 610041, China.
*Corresponding Authors：
Lian Li, E-mail: liliantripple@163.com;
Yuan Huang, E-mail: huangyuan0@163.com;

Experimental details

Materials:

Doxorubicin hydrochloride (DOX·HCl), 4-chlorobenzenesulfonate salt (DiD) were purchased from Dalian Meilun Biotech Co (Dalian, China). 3-Trimethyladenine, Rapamycin were purchased from Macklin (Shanghai, China). 3-(4, 5-dimethyl-2-tetrazolyl)-2,5-diphenyl-2H tetrazoliumbromide (MTT), 4′,6-diamidino-2-phenylindole (DAPI) were purchased from Sigma-Aldrich (St. Louis, MO, USA).

Antibody: anti-HMGB1 were purchased from Beyotime Biotechnology (Shanghai, China). Anti-calreticulin were purchased from (Abcam, material number: ab2907), anti-C/EBP homologous protein (CHOP) (Cell Signaling Technology, material number: 2895), anti-glucose regulated protein GRP78 (Beyotime Biotechnology, material number: AF0171). Anti-PI3K, anti-AKT, anti-phospho-AKT were purchased from HUABIO (Hangzhou, China), anti-phospho-PI3K, anti-AMPK,

anti-phospho-AMPK, anti-mTOR, anti-phospho-mTOR, anti-p62 were purchased from Wanleibio (Shenyang, China). Anti-LC3B (Cell Signaling Technology, material number: 43566S). Alexa Fluor 647-conjugated second antibody were purchased from Abcam (Cambridge, UK). CD8-depletion antibody was purchased from BD Biosciences (Bedford, USA). The primary antibodies against E-cadherin, the rabbit anti-actin primary antibodies and HRP-labelled goat anti-rabbit secondary antibodies were purchased from HUABIO (Hangzhou, China).

Cells and animals:

The 4T1 mouse mammary cancer cell line was acquired from Icell Biotechnology Co., Ltd. (Shanghai, China). 4T1 cells were grown in RPMI 1640 (Gibco, Invitrogen) media supplemented with 10% fetal bovine serum and 1% antibiotics (penicillin and streptomycin). These cells were incubated at 37°C with 5% CO_2 ($v\ v^{-1}$) in a thermostatic cell incubator. The BALB/c mice were obtained from Dashuo Biological Technology in Chengdu, China. Age-matched (6 to 8 weeks) female mice were utilized in all tests. The Medical Ethics Committee at Sichuan University approved all animal protocol research.

Synthesis and characterization of ED:

For the synthesis of ED, Dox· HCl (60.0mg, 0.103mol) was dissolved in DMF(1 mL). Then *p*-methyl benzene sulfonyl chloride (39.45mg, 0.12 mmol) in DMF(1 mL) were added to the solution, which reacted for 6 hours at 0 °C. The precipitation of crude ED was achieved through the use of diethyl ether. The resulting product was subsequently subjected to refinement via column chromatography, utilizing an eluent

composed of a mixture of DCM and MeOH in a ratio of 40:1. The structure of ED was determined through the application of nuclear magnetic resonance hydrogen spectrum (^{1}H NMR) analysis and mass spectrometry.

Investigation of accumulated autophagosomes after ED treatment

the numbers of autophagosomes in 4T1 cells were investigated by immunofluorescence. 4T1 cells were treated with ED (5 μg mL^{-1}, 10 μg mL^{-1}, 20 mL^{-1}) for 24 h after seeding 6 × 10^5 4T1 cells in 12-well plates overnight. After incubation with LC3B primary antibody overnight and AF647-labelled goat anti-rabbit secondary antibody for one hour, cells were washed three times by PBS, fixed by 4% polyformaldehyde, and stained with DAPI (5 μg mL^{-1}) for 5 min. Cells were imaged by CLSM to observe autophagosomes.

Investigation of autophagy induction:

The expression of LC3B, p62, PI3K, p-PI3K, AKT, p-AKT, AMPK, p-AMPK, mTOR, p-mTOR in 4T1 cells were measured by western blotting. 4T1 cells were treated with ED (5 μg mL^{-1}) or autophagy modulators (70 μM AP^{I} or 10 μM AP^{E}) for 24 h after seeding 1 × 10^6 4T1 cells in 6-well plates overnight. The cytosolic fractions were separated during 10 minutes of centrifugation at 10,000 rpm. The resulting cytosolic fractions were put onto sodium dodecyl sulfate-polyacrylamide gel electrophoresis (15% acrylamide). After SDS-PAGE separation, the proteins were transferred to a PVDF membrane (0.22 m, Keygen Biotechnology, Nanjing, China) and treated with LC3B and p62-specific primary antibodies. The proteins were then treated with secondary antibodies, and the chemiluminescence was assessed using a

Bio-Rad ChemDoc XRS System (BioRad, USA). The semi-semiquantitative results were analyzed by image J. Also, the numbers of autophagosomes in 4T1 cells were investigated by immunofluorescence. 4T1 cells were treated with ED (5 μg mL^{-1}) or autophagy modulators (70 μM AP^I or 10 μM AP^E) for 24 h after seeding 6×10^5 4T1 cells in 12-well plates overnight. After incubation with LC3B primary antibody overnight and AF647-labelled goat anti-rabbit secondary antibody for one hour, cells were washed three times by PBS, fixed by 4% polyformaldehyde, and stained with DAPI (5 μg mL^{-1}) for 5 min. Cells were imaged by CLSM to observe autophagosomes.

In vitro cytotoxicity investigation:

The evaluation of the in vitro cytotoxicity of two combinations against 4T1 cells was performed using 3-(4,5-Dimethylthiazol-2-yl)-2,5-diphenyltetrazolium bromide (MTT) assay. To initiate the experiment, 5×10^3 cells of 4T1 were seeded in each well of a 96-well plate and were allowed to incubate overnight. Following this, the cells were subjected to treatment with AP^I (70 μM) and AP^E (10 μM) in combination with a series concentrations of ED on 4T1 cells for 24 h. A solution of MTT (200 μL, 5 mg mL^{-1}) was added to each well, and subsequently incubated for a period of 4 hours. The supernatant was discarded and the remaining formazan crystals were dissolved with 150 μL of dimethyl sulfoxide (DMSO). The absorbance was measured using Varioskan Flash multimode reader at a wavelength of 490 nm (Thermo scientific, USA). The cell viability was calculated using the following formula: Cell

viability = ($OD_{490\ sample}$ – $OD_{490\ blank}$) / ($OD_{490\ control}$ – $OD_{490\ blank}$) × 100%. IC_{50} values were determined by GraphPad Prism 9.0 software.

Preparation, characterization and drug release of co-loaded nanoparticles:

Nanoparticles were synthesized using the widely employed nanoprecipitation technique. Poly-lactic-co-glycolic acid (50μl, 20 mg mL^{-1}), distearoylphosphoethanolamine-poly (ethylene glycol) (20μl, 20 mg mL^{-1}), and ED (100 μg, 0.14 mol), AP^{I} (160 μg, 1.07 mol) or AP^{E} (40 μg, 0.04 mol) were dissolved in DMSO, while soybean phospholipids (20μl, 10 mg mL^{-1}) were dissolved in methanol and DMSO in a 1: 1 mixture. Deionized water was gradually combined with the resulting organic mixture, which contained polymers, lipids, and medication, for 10 minutes. Dynamic light scattering was used to measure the nanoparticles' size distribution and zeta potential (DLS). Using ultraviolet spectroscopy GENESYS 180, the amount of drug that was encapsulated (We) and the total amount of drug (Wt) were calculated (Thermo Fisher Technologies, USA). To calculate the overall weight, the nanoparticle solution was thoroughly dehydrated (Wd). The formulas EE = We/Wt × 100% and DL = We/Wd × 100% were used to compute the entrapment efficiency (EE) and drug loading capacity (DL). After that, the nanoparticles were put in a dialysis bag (MWCO 3500 Da) and incubated in 50 mL of PBS with various pH levels (pH 7.4 or 6.5). 1 mL of the solution outside the dialysis bag was taken out and replaced with an equal volume of brand-new media at each predefined time interval. Thermo Scientific's Varioskan Flash 902-ULTS (Ex = 494 nm; Em = 587 nm, USA) was used to calculate the release of ED.

In vivo distribution of nanoparticles:

Mice were intravenously administered either DiD or DiD@NP when tumors grew to a size of 200 mm^3 (125 μg kg^{-1} equivalent DiD dosage, n = 3). The IVIS Spectrum In Vivo Imaging System was used to take pictures of mice at set intervals (PerkinElmer, Lumina 3, USA). Mice were sacrificed after receiving therapy for 24 h, and the same procedures were followed to collect tumor and major organ tissues for *ex vivo* fluorescence imaging.

In vitro anti-metastasis effect:

Wound healing, migration assays, and invasion assays were employed to investigate the impact of anti-metastasis in vitro. To perform the wound healing experiment, 4T1 cells were seeded onto 24-well plates and subjected to treatment with various drugs with an equivalence DOX dose of 2.5 μg mL^{-1} and equivalence AP^I dose of 70 μM or equivalence AP^E dose of 70 μM for a period of 24 hours. The distance migrated was determined by measuring the wounds at 0 h and 24 h at the same scratched place, and subsequently estimated using Image J software. In the migration test, 1×10^5 4T1 cells were seeded into the top chamber of transwell inserts. Following a 4-hour incubation period, the upper medium was replaced with various drug with an equivalence DOX dose of 2.5 μg mL^{-1} and equivalence AP^I dose of 70 μM or equivalence AP^E dose of 70 μM for an additional 24 hours. Inserts for transwell plates were fixed with 4% paraformaldehyde and then stained with 0.1% crystal violet solution. The cells that remained in the top chamber were eliminated, and the cells that migrated to the lower membranes were visualized under a

microscope (Leica Microsystems, Wetzlar, Germany) and dissolved in 33% acetic acid aqueous solution for quantitative analysis by Varioskan Flash at 590 nm (Thermo Scientific Varioskan Flash, Waltham, MA, USA). Additionally, Matrigel (BD Biosciences, San Diego, CA, USA) was applied to the inner bottom of the chamber 4 hours prior to the seeding of 4T1 cells, and the invasion test was performed in a manner similar to the migration assay.

In vitro anti-metastasis mechanism investigation:

The expression of the metastasis-associated proteins (SNAI1, vimentin) and autophagy-related proteins (LC3B, p62) were measured by western blotting. The expression of E-cadherin was analyzed using flow cytometry. Briefly, 4T1 cells were seeded onto 12-well plates and subjected to treatment with various drugs with an equivalence DOX dose of 2.5 μg mL^{-1} and equivalence AP^I dose of 70 μM or equivalence AP^E dose of 70 μM for a period of 24 h. After incubation, cells were harvested and followed by fixation with 4% paraformaldehyde for 15 min. Then the cells were incubated with anti-E-cadherin antibody in 1% BSA for 30 min at 4 °C and were subsequent stained with AF647-labelled goat anti-rabbit secondary for 1 h at 4 °C, followed by flow cytometry analysis.

In vivo anti-metastasis efficacy:

4T1 cells (3×10^4 cells) were injected into the third mammary fat pad of BALB/c female mice on day 0. When tumor volume reached 100 cm^3 on day 7, mice were intravenously injected with saline, AP^I@NPs, AP^E@NPs, ED@NPs, (ED+AP^I)@NPs, and (ED+AP^E)@NP with an equivalence DOX dose of 5 μmol kg^{-1} and equivalence

AP^I dose of 122 μmol kg^{-1} or equivalence AP^E dose of 17.4 μmol kg^{-1} every 3 days. On day 21, tumors were sacrificed, the tumor issue and lungs of each group were collected. Lungs from mice of all groups were fixed with Bouin's solutions for 4 h and then the metastatic nodules were counted. The expression of the metastasis-associated proteins (SNAI1) and autophagy-related proteins (LC3B, p62) were measured by western blotting. Tumor issues were fixed in 4% paraformaldehyde for at least 48 h and embedded in paraffin immunohistochemistry analysis of LC3B, p62, and E-cadherin.

In vivo anti-tumor efficacy, survival and long-term immune surveillance:

4T1 cells (3 ×10^4 cells) were injected into the third mammary fat pad of BALB/c female mice on day -7. When tumor volume reached 100 cm^3 on day 0, mice were intravenously injected with saline, AP^I@NPs, AP^E@NPs, ED@NPs, (ED+AP^I)@NPs, and (ED+AP^E)@NPs with an equivalence DOX dose of 5 μmol kg^{-1} and equivalence AP^I dose of 122 μmol kg^{-1} or equivalence AP^E dose of 17.4 μmol kg^{-1} every 3 days. The tumor size and body weight were recorded, and survival situations were recorded. The major organs were collected and stained with H&E (hematoxylin and eosin) for safety analysis.

In vivo CD8 depletion assay:

4T1 cells (3 ×10^4 cells) were injected into the third mammary fat pad of BALB/c female mice on day -7. When tumor volume reached 100 cm^3 on day 0, mice were intravenously injected with saline, (ED+AP^I)@NPs, (ED+AP^E)@NPs, companied with/without CD8 depleting antibodies (100 μg per mouse in sterile saline) per 3 days,

with an equivalence DOX dose of 5 μmol kg^{-1} and equivalence AP^I dose of 122 μmol kg^{-1} or equivalence AP^E dose of 17.4 μmol kg^{-1}. The tumor volumes of mice were measured every other day. On day 18, mice were sacrificed and the tumor issues were collected to weigh. Lungs were also collected and fixed with Bouin's solutions to count the metastatic nodules.

In vitro ER targeting efficiency:

In vitro ER dysfunction effect:

In order to investigate the effects of ER damage, the cells were seeded on 12-well plates with 6 × 10^5 4T1 cells and grown for 24 h. Following this, cells were treated with various drugs with an equivalence DOX dose of 5 μg mL^{-1} and equivalence AP^I dose of 70 μM or equivalence AP^E dose of 70 μM for a period of 24 h. Then the cells were incubated with primary antibodies against CHOP, GRP78, and elf2 in 1% BSA for 1 h at 4 °C, and were subsequent stained with AF647-labelled goat anti-rabbit secondary for 1 h at 4 °C, followed by flow cytometry analysis. For the research of cellular Ca^{2+} levels, 6 × 10^5 4T1 cells were seeded on 12-well plates overnight, subjected to various treatments (with an equivalence DOX dose of 5 μg mL^{-1} and equivalence AP^I dose of 70 μM or equivalence AP^E dose of 70 μM) for 12 h, stained with Fluo-4 AM (Cat No. 40704ES50; Yeasen, Shanghai, China) for 1 h, then incubated with medium for 30 min, and then subjected to flow cytometry analysis.

In vitro and in vivo ICD induction effect:

6×10^5 4T1 cells were seeded on 12-well plates, and the cells were cultured for 24 hours. Following this, the cells were treated with various drugs with an equivalence DOX dose of 5 μg mL^{-1} and equivalence AP^I dose of 70 μM or equivalence AP^E dose of 70 μM for a period of 24 h. Cells were harvested and washed with PBS, and incubated with anti-CRT primary antibody in 1% BSA for 1 h at 4 °C. Then Alexa Fluor647-labeled goat-anti-rabbit secondary antibody was added, the surface CRT on cells was detected by flow cytometry. To quantify *in vitro* ATP secretion and HMGB1 release, 6×10^5 4T1 cells were seeded on 12-well plates, and the cells were cultured for 24 h. Following this, the cells were treated with various drugs with an equivalence DOX dose of 5 μg mL^{-1} and equivalence AP^I dose of 70 μM or equivalence AP^E dose of 70 μM for a period of 24 h. Cell supernatants were collected and were and tested via ATP Elisa kits. The cellular and extracellular HMGB1 levels in treated cells were determined by western blotting. As for *in vivo* ICD induction capability, the 4T1 cells (3 ×104 cells) were injected into the third mammary fat pad of BALB/c female mice and were intravenously injected with saline, AP^I@NP, AP^E@NP, ED@NP, (ED+AP^I)@NP, and (ED+AP^E)@NP with an equivalence DOX dose of 5 μmol kg^{-1} and equivalence AP^I dose of 122 μmol kg^{-1} or equivalence AP^E dose of 17.4 μmol kg^{-1} every 3 days when tumor volume reached 100 cm^3. After various days, the tumors in each groups were collected and the tumor interstitial fluid was obtained by grinding the tumor tissues. The same method as *in vitro* assays was used to determine CRT levels in tumor issues. The tumor interstitial fluid were

collected to investigate ATP and HMGB1 levels by ATP Elisa kits and HMGB1 Elisa kits.

In vivo immune status investigation:

4T1 cells (3 ×10^4 cells) were injected into the third mammary fat pad of BALB/c female mice on day 0. When tumor volume reached 100 cm^3 on day 7, mice were intravenously injected with saline, AP^I@NP, AP^E@NP, ED@NP, (ED+AP^I)@NP, and (ED+AP^E)@NP with an equivalence DOX dose of 5 μmol kg^{-1} and equivalence AP^I dose of 122 μmol kg^{-1} or equivalence AP^E dose of 17.4 μmol kg^{-1} every 3 days. The mice were sacrificed on day 14 and the tumor issues were collected. To assess ICD induction, cells in tumor issues were stained with anti-CD45-PerCP/Cy5.5 and anti-CRT- antibodies for 1 hour at 4 °C. The cells were then washed and stained for 45 minutes with a second Alexa Fluor 647-conjugated secondary antibody. The cells were then cleaned before being analyzed by flow cytometry. To assess the infiltration of T lymphocytes in the tumor issues, cells in tumor issues were treated with anti-CD16/32 antibodies at 4 °C for 20 minutes, then stained with anti-CD3-FITC, anti-CD8-APC, and anti-CD4-PerCP/Cy5 antibodies to measure cytotoxic T lymphocytes ($CD3^+CD4^-CD8^+$) and T effector cells ($CD3^+CD4^+CD8^-$) in tumor issues. The cells were cleaned before being subjected to a flow cytometry analysis.

Statistical analysis:

Results were presented as mean ± standard deviations (SD). Statistical analysis was calculated by one-way ANOVA analysis using SPSS 22.0 software. P value <0.05 was recognized as statistically significant.

Supporting figures

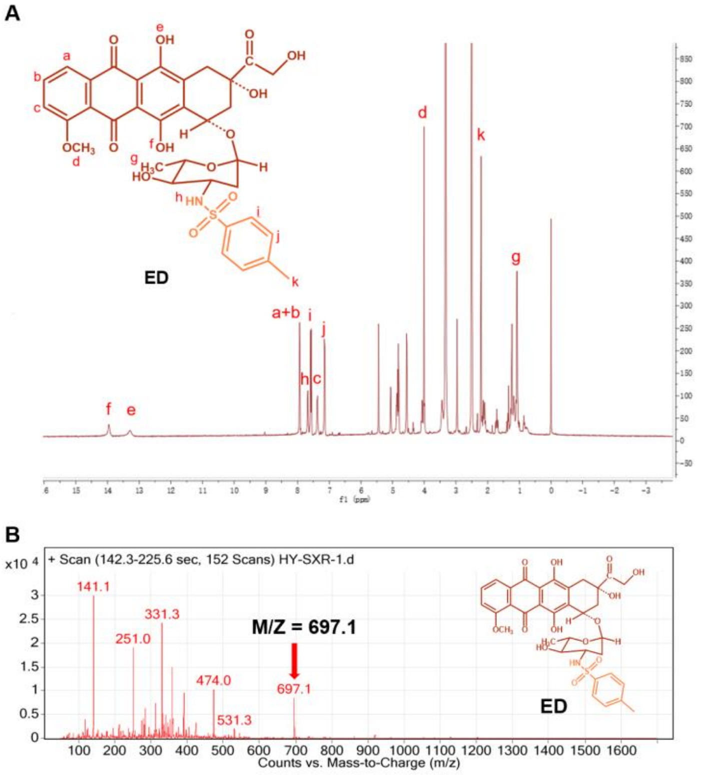

Figure S1: (1) ^{1}H NMR spectrum of ED in DMSO, a~g indicate the corresponding representative group magnetic displacement. (2) LC-MS spectrum of ED.

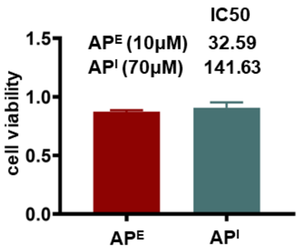

Figure S2: Cell viability results and IC_{50} of autophagy inducer rapamycin (AP^E) and autophagy inhibitor 3-MA (AP^I) for 4T1 cells at concentrations of 10 μM and 70 μM. Data was presented as mean ± SD. n = 5.

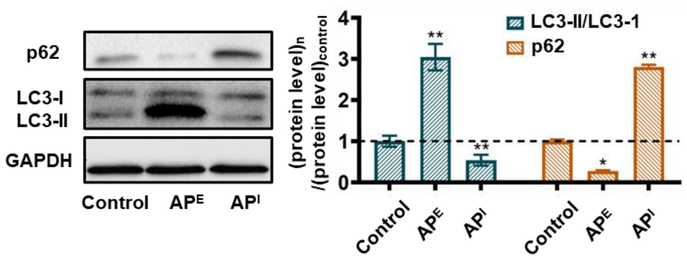

Figure S3: The protein level of LC3B analyzed in 4T1 cells after AP^E and AP^I treatment by western blotting. Data was presented as mean ± SD. n = 3. p*＜0.05, p**＜0.0 vs control.

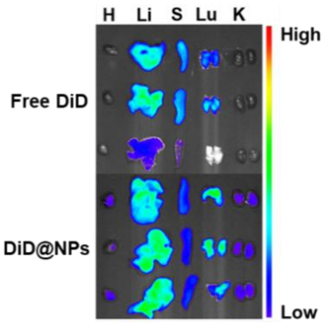

Figure S4: Images of the excised major organs at 24 h post-injection (n = 3).

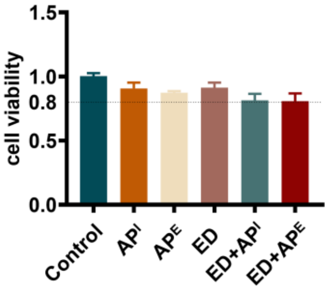

Figure S5: Cell viability of API (70 μM), APE (70 μM), ED (2.5 μg mL-1), and their combined groups on 4T1 cells for 24 h, respectively. The black dashed line represented the position of 80% cell viability. Data was presented as mean ± SD. n = 3.

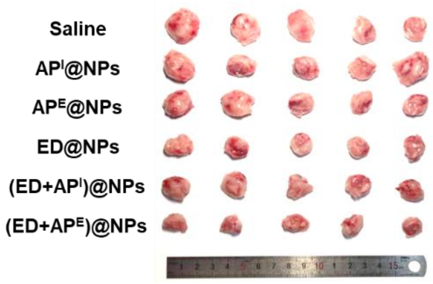

Figure S6: Representative images of the excised tumor tissues (n = 5).

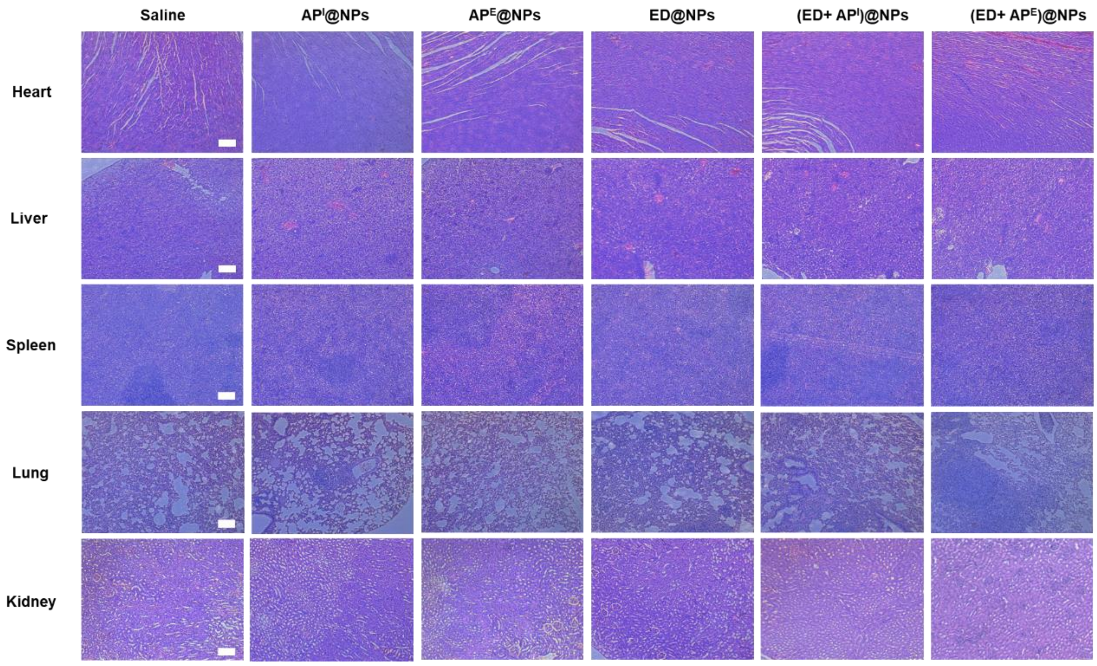

Figure S7: H&E staining of major organs from mice of each group (Scale bar = 200 μm).

Table S1. drug loading capacity (DL%) of different nanoparticles

	ED DL (%)	AP^I DL (%)	AP^E DL (%)
(ED+AP^I)@NPs	3.9	1.8	/
(ED+AP^E)@NPs	5.9	/	4.4
